# Time-restricted feeding rejuvenates cerebrovascular function and preserves cognition during aging

**DOI:** 10.21203/rs.3.rs-9520035/v1

**Published:** 2026-06-02

**Authors:** Madison Milan, Sharon Negri, Rakesh Rudraboina, Eva Troyano-Rodriguez, Jennifer Ihuoma, Aleksandra Kosmider, Shantipriya Awasthi, Woncheol Jung, Hassan Abushukair, Rohan Varshney, Greg Mullen, Raghavendra Yelahanka Nagaraja, Niharika Uppari, Mohiuddin Ahmad, Priya Balasubramanian, Shannon Conley, Andriy Yabluchanskiy, Anna Csiszar, Zoltan Ungvari, Audrey Cleuren, Michael Rudolph, Mickael Tanter, Tae Gyu Oh, Rafael de Cabo, Stefano Tarantini

**Affiliations:** 1) Vascular Cognitive Impairment and Neurodegeneration Program, Reynolds Oklahoma Center on Aging/Center for Geroscience and Healthy Brain Aging, Department of Neurosurgery, University of Oklahoma Health Science, Oklahoma City, Oklahoma, USA; 2) Stephenson Cancer Center, University of Oklahoma Health Science, Oklahoma City, Oklahoma, USA; 3) Oklahoma School of Science and Math, Oklahoma City, OK; 4) Department of Oncology Science, University of Oklahoma Health Science, Oklahoma City, OK; 5) Department of Biochemistry and Physiology, University of Oklahoma Health Science, Oklahoma City, OK; 6) Department of Cell Biology, University of Oklahoma Health Science, Oklahoma City, Oklahoma; 7) International Training Program in Geroscience, Doctoral College, Health Science Division/Institute of Preventative Medicine and Public Health, Semmelweis University, Budapest, Hungary; 8) Cardiovascular Biology Research Program, Oklahoma Medical Research Foundation; 9) Institute Physics for Medicine Paris, INSERM U1273, ESPCI PSL Paris, CNRS UMR 8631, PSL Research University, Paris, France; 10) Translational Gerontology Branch, Intramural Research Program, National Institute on Aging, National Institutes of Health, Baltimore, MD, USA

**Keywords:** Aging, Time-restricted feeding, Cerebrovascular aging, Neurovascular coupling, Blood–brain barrier, Endothelial metabolism, Mitochondrial function, Metabolic reprogramming, Ketone bodies, Cognitive decline

## Abstract

Cerebromicrovascular dysfunction is a key driver of age-related cognitive decline, yet interventions targeting microvascular aging remain limited. Here, we show that time-restricted feeding (TRF) preserves cognitive function and rejuvenates cerebrovascular physiology in aged mice. TRF improves resting cerebral blood flow and neurovascular coupling while attenuating blood–brain barrier disruption, neuroinflammation, and endothelial senescence. Mechanistically, TRF enhances metabolic flexibility and restores mitochondrial bioenergetic capacity in cerebromicrovascular endothelial cells. Ketone bodies elevated by TRF recapitulate key mitochondrial and vascular effects, improving endothelial respiration, membrane potential, and redox balance in aged mice and primary human brain endothelial cells, but do not fully reproduce neurovascular unit protection. These findings identify endothelial mitochondrial reprogramming as a central mechanism linking dietary timing to cerebrovascular resilience and cognitive preservation, and suggest that metabolic interventions can partially reverse key features of vascular brain aging.

Microvascular dysfunction is increasingly recognized as a fundamental contributor to age-related cognitive decline and dementia [1]. Beyond large-vessel pathology, alterations in the cerebral microcirculation, including impaired neurovascular coupling (NVC), reduced capillary density, and disruption of blood–brain barrier (BBB) integrity, play a central role in limiting oxygen and nutrient delivery to active brain regions and in promoting neuroinflammation [2–4]. These microvascular deficits are now understood to precede and exacerbate neuronal dysfunction, positioning the cerebral microvasculature as a critical and potentially modifiable driver of cognitive aging [5–7]. However, whether cerebrovascular aging can be actively reversed, rather than simply slowed down, remains unclear, and effective interventions targeting microvascular function are lacking. The integrity of the cerebral microvasculature is critically dependent on endothelial cell function, which orchestrates vascular tone, barrier properties, and neurovascular signaling within the neurovascular unit (NVU). With aging, endothelial cells undergo a progressive functional decline marked by reduced nitric oxide bioavailability, increased oxidative stress, and accumulation of senescent phenotypes [8–12]. Mitochondrial dysfunction has emerged as a central mechanism underlying these changes [13]. Impaired mitochondrial respiration and reduced bioenergetic flexibility drive redox imbalance and inflammatory signaling, thereby disrupting NVC and BBB integrity. Consequently, these observations position endothelial mitochondrial function as a central and potentially tractable regulator of cerebrovascular aging [14], linking systemic metabolic state to neurovascular unit integrity and cognitive outcomes.

Systemic metabolic interventions have emerged as powerful modulators of aging biology, with growing evidence that targeting metabolic flexibility can influence vascular and brain function [15–18]. Time-restricted feeding (TRF), a dietary approach that encourages eating within a set time frame in alignment with circadian rhythms without limiting calorie intake, induces recurring cycles of metabolic switching between glucose and lipid utilization. This transition is accompanied by increased production of circulating ketone bodies, particularly β-hydroxybutyrate (β-HB), which functions both as an energy substrate and as a signaling metabolite [19, 20]. β-HB has been shown to regulate oxidative stress, inflammation, and mitochondrial function across multiple cell types [21–23]. Importantly, endothelial cells exhibit sensitivity to TRF [24], and ketone-mediated modulation of mitochondrial bioenergetics and redox homeostasis within the cerebrovasculature may provide a mechanistic link between systemic metabolic state and microvascular function [25]. Despite a growing interest in metabolic interventions, the extent to which TRF modulates cerebrovascular aging, and whether these effects are mediated by endothelial metabolic reprogramming, remains unclear. In particular, it is not known whether TRF-induced metabolic switching and ketone body production can preserve neurovascular unit integrity and mitigate cognitive decline during aging.

Here, we tested the hypothesis that TRF attenuates age-related cognitive decline by preserving cerebrovascular function through improvements in endothelial mitochondrial bioenergetics. Using a combination of *in vivo* functional imaging, behavioral analyses, and mechanistic studies in both murine models and primary human cerebromicrovascular endothelial cells (HCMVECs), we show that TRF maintains neurovascular coupling, preserves BBB integrity, and enhances endothelial mitochondrial function. Furthermore, we identify circulating ketone bodies as key mediators linking systemic metabolic adaptation to cerebrovascular resilience. Together, these findings identify endothelial mitochondrial reprogramming as a central mechanism linking dietary timing to cerebrovascular resilience and suggest that key features of vascular brain aging are metabolically plastic.

## Results

### Time-restricted feeding prevents age-related deficits in hippocampal-dependent spatial learning and memory

**Figure 1.**
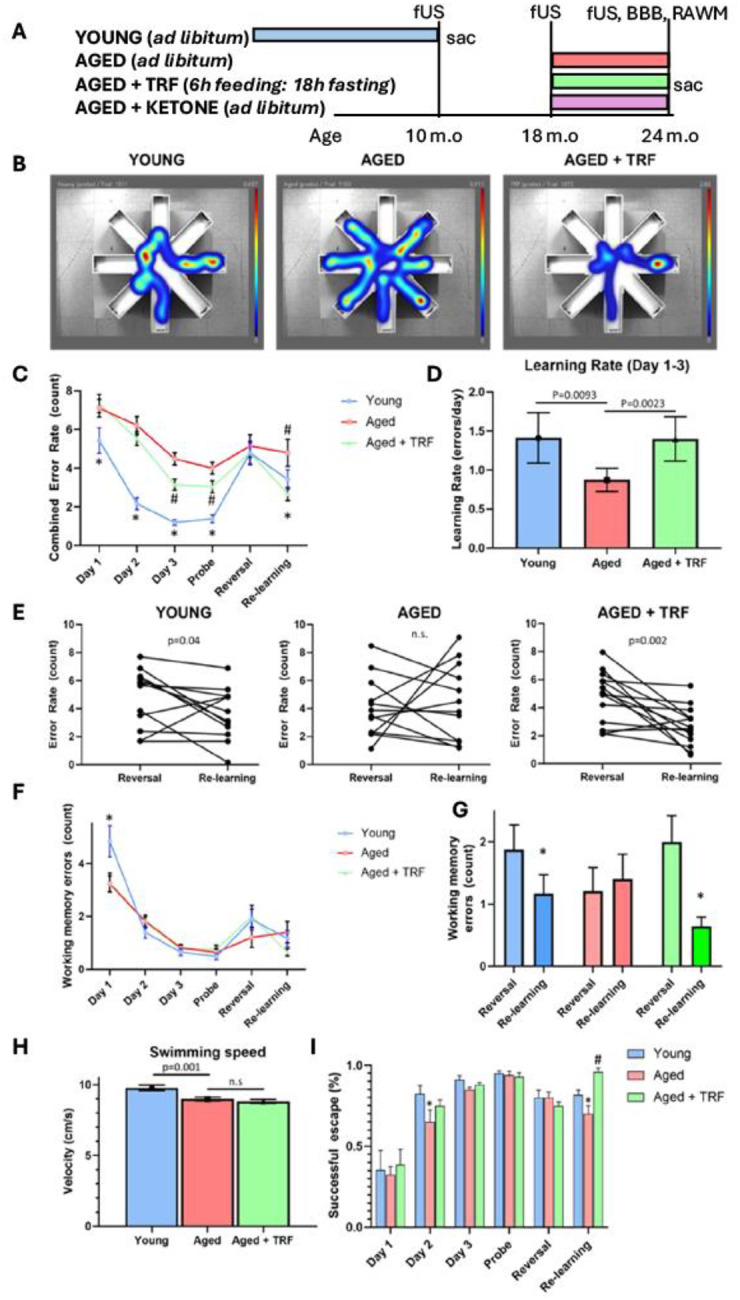
TRF preserves hippocampal-dependent spatial learning and memory in aging. **A)** Graphical representation of the study design. **B)** Representative heat maps depicting the movement of an animal in each group during a trial of radial arm water maze. **C)** Combined error rate during each phase of the behavioral testing, which is calculated by adding 1 error for each incorrect arm entry as well as for every 15 seconds spent not exploring the arms. **D)** Learning rate is characterized by slope of the number of errors during the learning phase (days 1–3). **E)** Comparison of the number of errors made by the same animals during the reversal and relearning phases. Connected data points represent a single animal during each of these phases. **F)** Comparison of the working memory by number of errors during each phase. **G)** Number of errors during the reversal and relearning phases for each group. **H)** Swimming speed during each group. **I)** Successful escapes from the maze performed by each group during each phase. All data are shown as mean ± SEM. (n=20 in each group). Statistical significance was calculated using one-way ANOVA with Tukey’s post hoc test to determine differences among groups. *p<0.05, **p<0.01, ***p<0.001, ****p<0.0001.

To evaluate the potential of fasting as an intervention against age-related cognitive decline, 18-month-old mice were placed on a TRF regimen for 6 months. Outcomes were compared to those of 10-month-old and 24-month-old control mice (Figure 1A). We first examined whether TRF attenuates age-related cognitive decline using the radial arm water maze (RAWM). Aged control mice exhibited significantly impaired spatial learning and memory compared to young controls, as indicated by increased error rates during both the acquisition and probe phases (Figure 1B–C). In contrast, aged mice subjected to TRF showed a marked improvement in performance, with reduced errors during the probe phase and enhanced learning rates (Figure 1 B–D). During reversal learning, aged TRF mice demonstrated improved working memory and adaptability, performing similarly to young controls (Figure 1E–G, I). Importantly, swimming speed did not differ between aged control and aged TRF mice, indicating that these effects were not attributable to changes in motor function (Figure 1H). Consistent with prior studies, TRF was also associated with improved survival in aged mice (Extended Figure 1). Together, these findings indicate that TRF preserves hippocampal-dependent learning and memory during aging, supporting a functional benefit on cognition independent of motor performance. These behavioral improvements align with converging evidence that dietary timing, independent of caloric reduction, represents a potent modulator of brain aging. Circadian alignment of feeding has recently been shown to extend healthy lifespan in mice beyond the effects of caloric restriction alone, with restoration of inflammation-associated gene expression patterns in aging liver tissue [26, 27]. Notably, in rodent models of Alzheimer’s disease, TRF improved hippocampal transcription, memory performance, and pathological hallmarks through broad transcriptional remodeling of vascular and inflammatory pathways [28], consistent with the multi-system cerebrovascular protection observed here. The preservation of reversal learning and working memory in aged TRF mice further suggests that TRF targets neural circuit flexibility, a cognitive domain particularly sensitive to prefrontal-hippocampal circuit integrity and microvascular perfusion. Given that NVC impairment in the frontal cortex independently predicts age-related cognitive dysfunction, the functional improvements observed here likely reflect both vascular and circuit-level rescue.

### Time-restricted feeding maintains vascular density across cortical and hippocampal regions during aging

**Figure 2.**
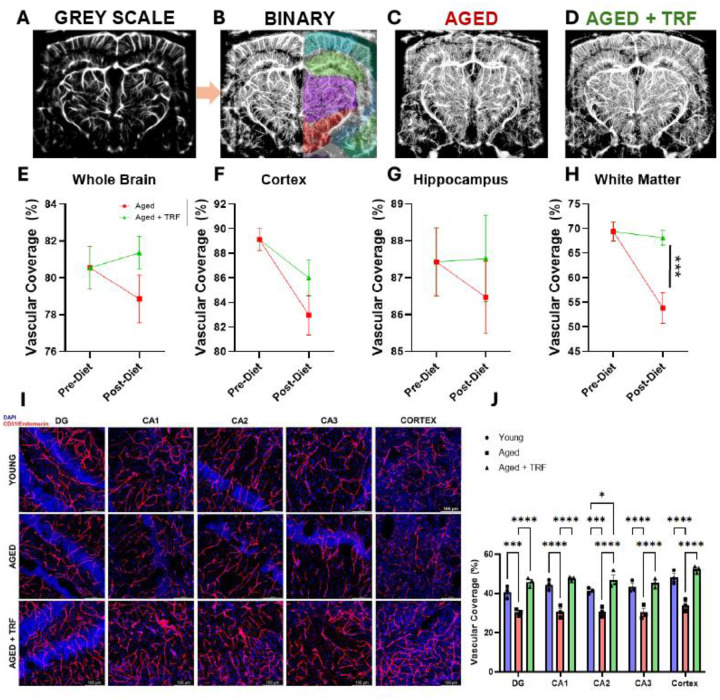
TRF delays vascular density loss across cortical and hippocampal regions during aging. **A-D)** Representative images of vascular density in the brain measured by ICONEUS functional ultrasound, which was binarized and overlayed with the Allen brain atlas to identify specific neuroanatomical regions. **E-H)** Vascular coverage in the whole brain, cortex, hippocampus, and white matter before starting TRF regimen (18 months of age) and at the experimental endpoint (24months of age) after 6 months of *ad libitum* diet for controls or TRF regimen for aged TRF mice. **I)** Representative confocal microscopic images of cortical and hippocampal regions in embedded brain sections stained with DAPI (blue) and CD31/Endomucin (red). **J)** Quantification of vascular coverage in cortex and hippocampus from immunohistochemical staining. All data are shown as mean ± SEM. Statistical significance was calculated using unpaired t tests **(E-H)** and two-way ANOVA with Tukey’s post hoc test to determine differences among groups **(J)**. *p<0.05, **p<0.01, ***p<0.001,****p<0.0001.

We next assessed whether TRF mitigates age-related cerebrovascular rarefaction using functional ultrasound imaging and ultrasound localization microscopy. Aged control mice exhibited a progressive decline in vascular density across multiple brain regions, including the cortex, hippocampus, and white matter (Figure 2A–H). In contrast, aged TRF mice showed preservation of vascular density, with significantly greater vascular coverage in white matter and trends toward protection in other regions (Figure 2A–H). The preferential protection of white matter macrovasculature by TRF is consistent with the known vulnerability of periventricular white matter to microvascular dysfunction in aging [29], and suggests that dietary timing interventions may be particularly effective in regions most susceptible to hypoperfusion-related injury. These findings were corroborated by immunohistochemical analyses, which demonstrated maintenance of microvascular density in cortical and hippocampal regions in TRF-treated mice, comparable to young controls (Figure 2I–J). These results indicate that TRF attenuates age-related microvascular rarefaction, a key structural determinant of impaired cerebral perfusion and cognitive decline. The magnitude of microvascular preservation observed here is particularly noteworthy given that age-related capillary rarefaction typically reduces vessel density by 15–25% in cortical regions by 24 months of age [30, 31], and this rarefaction is strongly correlated with reduced cognitive performance in aged mice [29].

### Time-restricted feeding maintains resting cerebral blood flow and neurovascular coupling in aging

**Figure 3.**
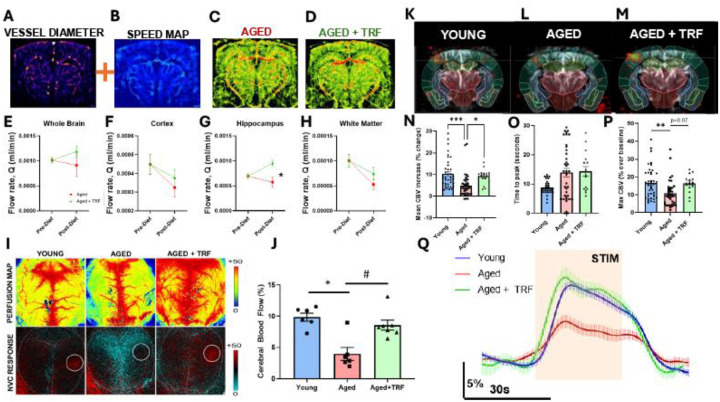
Time-restricted feeding maintains resting cerebral blood flow and neurovascular coupling in aging. **A-D)** Representative image of the resting CBF in the brains of aged and aged TRF mice using ICONEUS functional ultrasound. **E-H)** Quantification of the flow rate in the whole brain, cortex, hippocampus, and white matter before starting TRF regimen (18 months of age) and at the experimental endpoint (24 months of age) after 6 months of *ad libitum* diet for controls and TRF regimen for aged TRF mice. **I)** Representative cerebral perfusion map using laser speckle contrast imaging (top). NVC responses (bottom) in contralateral somatosensory whisker barrel cortex (white circle) after whisker stimulation. **J)** Cerebral blood flow after whisker stimulation as a percent of the baseline CBF. **K-M)** Representative images of CBF to somatosensory whisker barrel cortex in response to whisker stimulation, measured by fUS. **N-Q)** NVC responses measured by fUS, indicated by increases in cerebral blood volume, time to maximum cerebral blood volume, and maximum cerebral blood volume. All data are shown as mean ± SEM. Statistical significance was calculated using unpaired t tests **(E-H)** and one-way ANOVA with Tukey’s post hoc test **(J, N-P)**. *p<0.05, **p<0.01, ***p<0.001, ****p<0.0001.

We next evaluated whether TRF preserves resting cerebral blood flow (CBF) and NVC. Aged control mice exhibited reduced resting CBF as compared to young controls, particularly in the hippocampus, whereas aged TRF mice maintained significantly higher flow rates (Figure 3A–H). In response to whisker stimulation, aged control mice showed impaired NVC, as evidenced by blunted increases in CBF (Figure 3I–Q). In contrast, TRF preserved these responses in aged mice, demonstrating NVC patterns comparable to young controls (Figure 3I–Q). Notably, long-term potentiation did not differ between aged control and aged TRF mice, suggesting that the observed improvements are primarily driven by vascular rather than synaptic mechanisms (Extended Figure 3). Collectively, these findings indicate that TRF preserves both basal and activity-dependent cerebrovascular function during aging.

### BBB integrity is protected by TRF regimen during aging, which reduces overall neuroinflammation and senescence

**Figure 4.**
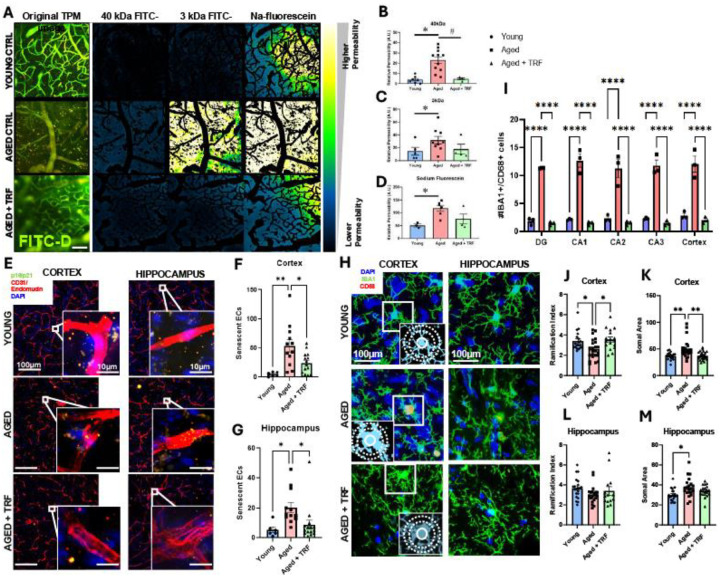
Blood-brain barrier integrity is protected by TRF regimen during aging, reducing neuroinflammation and senescence. **A)** *In vivo* two-photon microscopy representative images of brain cortex after retroorbital injection of fluorescent tracers. Left panel: Glycocalyx stained with wheat germ agglutinin (WGA). Right panels: Injection of fluorescent tracers (40kDa, 3kDa, and 0.3kDa/sodium fluorescein) showing progressive extravasation from the vessel. **B-D)** Quantification of relative permeability of 40kDa, 3kDa, and 0.3kDa tracers. **E)** Representative images of cortical and hippocampal sections stained with CD31/endomucin vascular markers and p16/p21 senescence markers. **F-G)** Quantification of p16+/p21+ endothelial cells in cortex and hippocampus. **H)** Representative confocal microscopy images of microglial morphology as indicated by sholl analysis. **I)** Quantification of IBA1+/CD68+ cells in cortical and hippocampal regions, representing activated microglia. **J-M)** Ramification index and somal size of microglia in the cortex and hippocampus of each group. All data are shown as mean ± SEM. Statistical significance was calculated using one-way ANOVA with Tukey’s post hoc test **(B-D, F, G, J)** or two-way ANOVA with Tukey’s post hoc test **(I)**. *p<0.05, **p<0.01, ***p<0.001, ****p<0.0001.

Because disruption of BBB integrity is a key consequence of cerebrovascular aging and an important contributor to neuroinflammation and cognitive decline, we next assessed whether TRF preserves barrier function *in vivo*. Aged control mice exhibited significantly increased microvascular permeability, as demonstrated by enhanced extravasation of fluorescent tracers across a range of molecular weights (Figure 4A–D). In contrast, aged TRF mice showed reduced tracer leakage, indicating maintained BBB integrity (Figure 4A–D). Consistent with these findings, immunohistochemical analyses revealed a marked reduction in senescent endothelial cells in the cortex and hippocampus of aged TRF mice compared to aged controls (Figure 4E–G), which is a known driver of BBB disruption [10]. In parallel, TRF attenuated age-related neuroinflammation, as evidenced by decreased microglial activation, reduced somal size, and preservation of microglial ramification patterns (Figure 4H–M). These observations were further supported by single-cell RNA sequencing, which demonstrated reduced expression of senescence-associated gene signatures across multiple neurovascular unit cell types in aged TRF mice relative to aged controls (Figure 8G–M). Together, these findings indicate that TRF preserves BBB integrity, and mitigates neuroinflammation and cellular senescence, key pathological features of cerebrovascular aging.

### Time-restricted feeding enhances metabolic flexibility in aging

**Figure 5.**
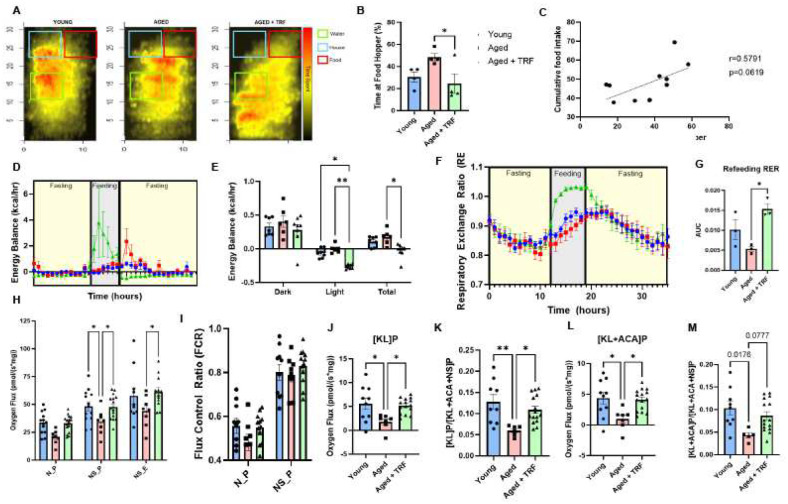
Time-restricted feeding enhances metabolic flexibility in aging. **A)** Representative heat map of the time spent in each part of the cage, relative to the water, house, and food. **B)** Quantification of the percent of time spent at the food hopper in each group. **C)** Correlation of the cumulative food intake with the amount of time spent at the food hopper among all groups. **D)** Hourly energy balance in fasting and feeding periods.**E)** Quantification of energy balance during dark and light cycles. **F)** Hourly respiratory exchange ratio in fasting and feeding periods. **G)** Area under the curve of respiratory exchange ratio during the refeeding period. **H)** Mitochondrial respiration in the brain cortex tissue during oxidative phosphorylation with complex I-linked substrates (N_P) and complex I- and II-linked substrates (NS_P), and respiration during the electron transfer state using complex I- and II-linked substrates (NS_E). **I)** Flux control ratio (FCR), or coupling efficiency, during oxidative phosphorylation with complex I-linked substrates (N_P) and complex I- and II-linked substrates (NS_P). **J)** Complete ketolytic respiration in the brain cortex. **K)** Ratio of complete ketolysis to OXPHOS. **L)** β-hydroxybutyrate and acetoacetate-linked respiration in the brain cortex. **M)** Ratio of ketone-linked respiration to OXPHOS. All data are shown as mean ± SEM. Statistical significance was calculated using one-way ANOVA with Tukey’s post hoc test **(B,G, J-M),** two-way ANOVA with Tukey’s post hoc test **(E, H, I),** or simple linear regression (C). *p<0.05, **p<0.01, ***p<0.001, ****p<0.0001.

We next examined whether TRF induces metabolic adaptations that could underlie its protective effects on the cerebrovasculature. Despite the lack of significant differences in total caloric intake or body mass, aged TRF mice exhibited distinct behavioral and metabolic patterns, including reduced time spent at the food hopper and lower overall energy balance (Figure 5A–E, Extended Figure 4). Importantly, TRF induced pronounced oscillations in respiratory exchange ratio, reflecting enhanced metabolic flexibility and dynamic shifts between fuel sources (Figure 5F–G). Consistent with these systemic changes, high-resolution respirometry of cortical tissue revealed that aged control mice exhibited impaired mitochondrial respiration, whereas aged TRF mice demonstrated significantly improved oxidative phosphorylation capacity and electron transport system activity (Figure 5H–I). Furthermore, TRF enhanced the capacity of cortical mitochondria to utilize ketone bodies, as evidenced by increased respiration under ketolytic conditions (Figure 5J–M). Together, these findings indicate that TRF promotes metabolic flexibility and enhances mitochondrial bioenergetic capacity in the aging brain. These findings indicate that TRF promotes metabolic flexibility and enhances mitochondrial bioenergetic capacity in the aging brain. The cyclic metabolic switching induced by TRF, alternating between glucose-based and lipid-based fuel utilization across the feeding and fasting phases, is consistent with the conceptual framework proposed by Mattson [27], in which recurring transitions between metabolic states drive adaptive cellular stress responses that enhance mitochondrial efficiency and reduce oxidative damage over time. The observation that cortical mitochondria from aged TRF mice exhibit enhanced ketolytic respiration is particularly noteworthy, as it indicates not only increased ketone body availability, but a coordinated upregulation of enzymatic machinery for ketone oxidation, including activity of β-hydroxybutyrate dehydrogenase and downstream TCA cycle engagement. These findings position TRF-induced metabolic reprogramming as a multi-level adaptation that encompasses both circulating substrate availability and intrinsic mitochondrial substrate utilization capacity, a distinction that has important implications for understanding why ketone supplementation alone does not fully recapitulate TRF’s effects. The absence of significant differences in total caloric intake between aged control and aged TRF mice confirms that the metabolic adaptations observed are driven by the temporal pattern of feeding rather than by caloric restriction per se, consistent with recent large-scale studies demonstrating that fasting duration and circadian alignment, rather than calorie reduction alone, are primary drivers of the geroprotective effects of dietary restriction.

### Fasting-induced circulating ketone bodies improve mitochondrial bioenergetic health in human cerebromicrovascular endothelial cells

**Figure 6.**
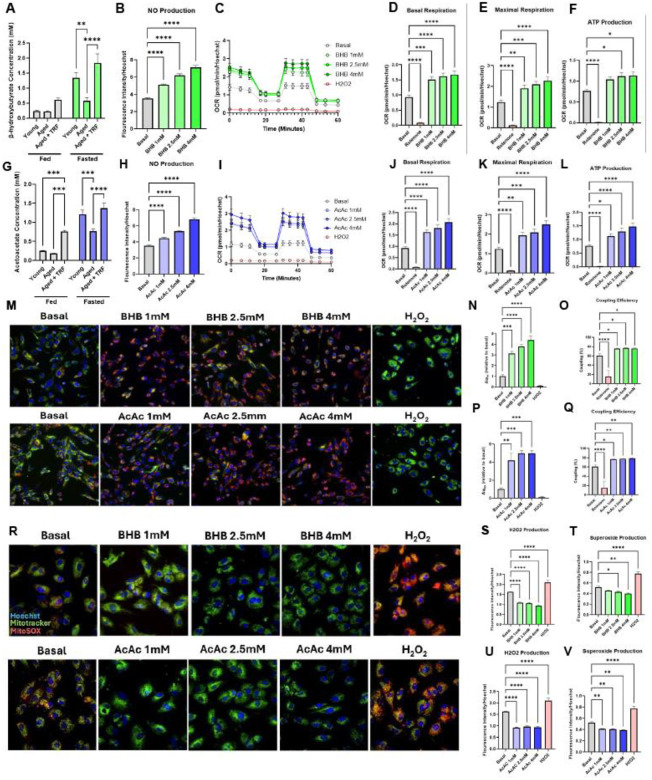
Fasting-induced circulating ketone bodies improve mitochondrial bioenergetic health in human cerebromicrovascular endothelial cells. **A, G)** Concentration of serum β-hydroxybutyrate and acetoacetate during the fed period and an acute fasting period in each group. **B, H)** Nitric oxide production after 24 hour treatment of HCMVECs with β-HB or AcAc. **C, I)** Oxygen consumption rate (OCR) during MitoStress test using Seahorse respirometry after 24 hour treatment of HCMVECs with β-HB or AcAc. **D-F, J-L, O, Q)** Basal respiration, maximal respiration, ATP production, and coupling efficiency measured using Seahorse respirometry after 24 hour treatment of HCMVECs with β-HB or AcAc. **M)** Representative images of JC-1 assay to measure mitochondrial membrane potential with HCMVECs treated with β-HB or AcAc. **N, P)** Quantification of mitochondrial membrane potential. **R)** Representative images of MitoSox assay to test mitochondrial superoxide production in HCMVECs treated with β-HB or AcAc. **S-V)** Quantification of superoxide production using MitoSox Red and hydrogen peroxide production using Amplex Red. All data are shown as mean ± SEM. Statistical significance was calculated using one-way ANOVA with Dunnett’s multiple comparisons (**B, D-F, H, J-L, N-Q, S-V**) or two-way ANOVA with Tukey’s post hoc test (**A, G**). *p<0.05, **p<0.01, ***p<0.001, ****p<0.0001.

TRF and acute fasting significantly increased circulating levels of β-hydroxybutyrate (β-HB) and acetoacetate (AcAc), with aged TRF mice exhibiting elevated ketone concentrations even in the fed state (Figure 6A, G). To determine whether ketone bodies directly contribute to the observed cerebrovascular benefits, we assessed their effects on human cerebromicrovascular endothelial cells. Treatment of endothelial cells with physiologically relevant concentrations of β-HB and acetoacetate [32] resulted in marked improvements in mitochondrial function, including increased basal and maximal respiration, enhanced ATP production, and improved coupling efficiency (Figure 6C–F, I-L, O, Q). In addition, ketone treatment increased mitochondrial membrane potential and reduced the production of reactive oxygen species, including superoxide and hydrogen peroxide (Figure 6 M, N, P, R-V). Nitric oxide production was also significantly increased, indicating improved endothelial function (Figure 6B, H). Together, these findings demonstrate that ketone bodies directly enhance mitochondrial bioenergetics and redox homeostasis in cerebrovascular endothelial cells, providing a mechanistic link between TRF-induced metabolic switching and vascular function.

### Ketone-rich diet alleviates age-related decline of mitochondrial and NVU function

**Figure 7.**
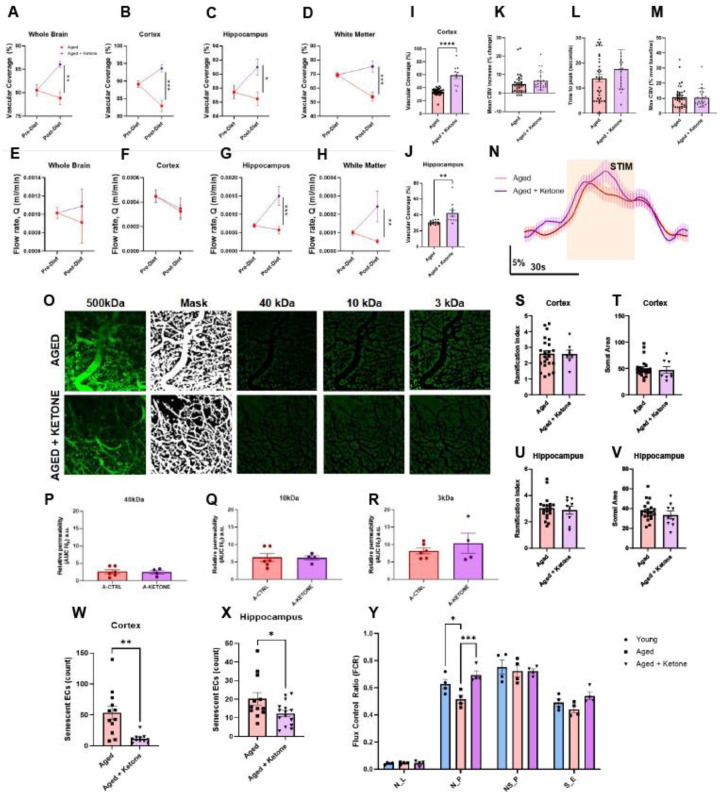
Ketone-rich diet alleviates age-related decline of mitochondrial and NVU function. **A-D)** Vascular coverage in the whole brain, cortex, hippocampus, and white matter before starting ketone-rich diet (18 months of age) and at the experimental endpoint (24 months of age) after 6 months of *ad libitum* diet for controls or ketone diet for aged ketone mice. **E-H)** Quantification of the flow rate in the whole brain, cortex, hippocampus, and white matter before starting ketone diet (18 months of age) and at the experimental endpoint (24 months of age) after 6 months of *ad libitum* diet for controls and ketone-rich diet for aged ketone mice. **I-J)** Vascular coverage in the cortex and hippocampus of aged control and aged ketone mice measured by immunohistochemical quantification. **K-N)** NVC responses measured by fUS, indicated by increases in cerebral blood volume, time to maximum cerebral blood volume, and maximum cerebral blood volume. **O)** *In vivo* two-photon microscopy representative images of brain cortex after retroorbital injection of fluorescent tracers. Left panel: Vessels are marked after retroorbital injection with 500kDa tracer, allowing for binarization for dynamic subtraction. Right panels: Injection of fluorescent tracers (40kDa, 10kDa, and 3kDa) showing progressive extravasation from the vessel. **P-Q)** Quantification of relative permeability of 40kDa, 10kDa, and 3kDa tracers. **S-V)** Ramification index and somal size of microglia in the cortex and hippocampus of each group, quantified from immunohistochemistry. **W-X)** Quantification of p16+/p21+ endothelial cells in cortex and hippocampus from immunohistochemistry. **y)** Flux control ratio (FCR), or coupling efficiency, during oxidative phosphorylation with complex I-linked substrates (N_P) and complex I- and II-linked substrates (NS_P).

We next assessed whether exogenous elevation of ketone bodies recapitulates the protective effects of TRF *in vivo*. Aged mice maintained on a ketone-rich diet exhibited significantly increased vascular density across multiple brain regions, including the cortex, hippocampus, and white matter (Figure 7A–D, I-J) as compared to regular diet fed mice. These structural improvements were accompanied by enhanced resting cerebral blood flow, particularly in hippocampal and white matter regions (Figure 7E–H). In contrast to TRF, ketone supplementation did not significantly improve neurovascular coupling or BBB integrity, nor did it alter microglial activation or morphology (Figure 7 K–V). However, the ketone-rich diet significantly reduced endothelial cell senescence and improved mitochondrial coupling efficiency in cortical tissue (Figure 7W–Y). Together, these findings indicate that ketone bodies partially recapitulate the mitochondrial and structural benefits of TRF, while suggesting that additional TRF-dependent mechanisms contribute to full neurovascular unit protection.

### Fasting transcriptome highlights an increased cell proportion of mitochondria-enriched endothelial cells in the brain cortex

**Figure 8.**
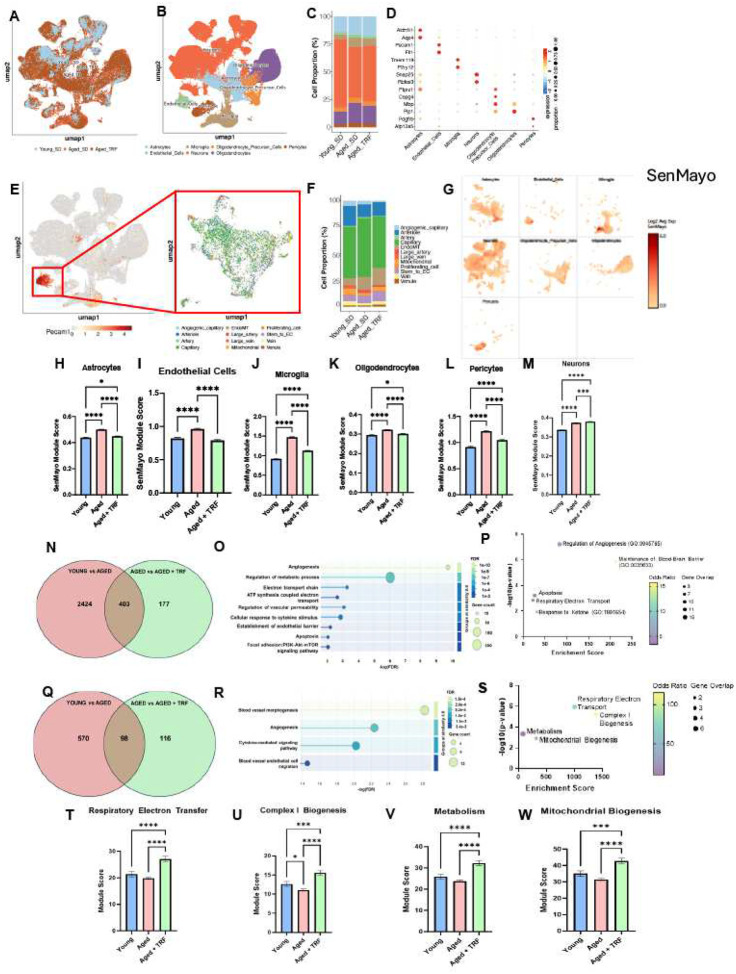
Fasting transcriptome highlights an increased cell proportion of mitochondria-enriched endothelial cells in the brain cortex. Single cell RNA sequencing (scRNAseq) was performed using brain cortex tissue from each group (n=7 young control, 14 aged control, 8 aged TRF; 208K total cells). **A)** UMAP of cells from each group. **B)** UMAP clustering of each cell type. **C)** Proportion of each cell type by group. **D)** Most highly expressed markers of each cell type. **E)** Endothelial cell population expressing PECAM1, with endothelial cell subpopulations being identified. **F)** Proportion of subpopulations of endothelial cells, with the mitochondria-active subpopulation indicated by the red box. **G)** UMAP of the expression of genes in the SenMayo gene set in each cell type. **H-M)** Expression of genes in the SenMayo gene set in each cell type in each group. **N)** Differentially expressed genes (DEGs) between young and aged endothelial cells compared to aged and aged TRF endothelial cells. **O-P)** Selected pathways significantly enriched in the overlapping portion of DEGs. **Q)** DEGs between young and aged compared to aged and aged TRF in the capillary sub-population of endothelial cells. **R-S)** Selected pathways significantly enriched in the overlapping portion of DEGs. **T-W)** Expression of genes overlapping with the respiratory electron transfer, complex I biogenesis, metabolism, and mitochondrial biogenesis pathways quantified in capillary subpopulation of endothelial cells in each group. Data are shown as mean ± SEM, with statistical significance calculated using one-way ANOVA with Tukey’s post hoc test. *p<0.05, **p<0.01, ***p<0.001, ****p<0.0001.

To characterize cell-type-specific responses to TRF, we performed single-cell RNA sequencing of cortical tissue across experimental groups. TRF was associated with a shift in endothelial cell populations, including an increased proportion of mitochondria-enriched endothelial subtypes (Figure 8A–F). Comparative transcriptomic analyses identified gene expression changes associated with aging that were reversed by TRF, with significant enrichment in pathways related to mitochondrial bioenergetics, vascular function, and inflammation (Figure 8N–P, Extended Figure 6). Similar patterns were observed within capillary endothelial subpopulations, where TRF enhanced the expression of genes involved in respiratory electron transport and mitochondrial biogenesis (Figure 8Q–W, Extended Figure 5). These findings indicate that TRF induces cell-type-specific transcriptional reprogramming within the cerebrovasculature, characterized by enhanced mitochondrial function and reduced inflammatory and senescence-associated signaling. These transcriptomic findings are of particular significance in light of recent single-cell atlases demonstrating that brain endothelial cells are highly heterogeneous, with functionally distinct arterial, capillary, and venous subpopulations exhibiting different susceptibilities to aging-related stress [12, 33]. The selective enrichment of mitochondria-active capillary endothelial cells by TRF is consistent with the known role of capillary endothelium as the primary site of NVC and metabolic exchange, and suggests that TRF selectively restores the functional identity of the cell population most critical for activity-dependent CBF regulation. The reversal of aging-associated transcriptional signatures, particularly in pathways governing electron transport chain assembly, mitochondrial biogenesis, and ROS handling, provides a molecular basis for the functional improvements observed at the tissue and behavioral levels. The reduction of senescence gene module scores across multiple NVU cell types (Figure 8G–M) further indicates that TRF does not only compensate for existing dysfunction, but may attenuate the accumulation of senescent cells in the cerebrovascular niche over time. These cell-type-specific transcriptional data provide a high-resolution molecular blueprint of TRF-mediated cerebrovascular rejuvenation, and identify the capillary endothelial mitochondrial bioenergetic network as the primary transcriptional target of dietary timing interventions in the aging brain. Together, these findings indicate that TRF induces cell-type-specific transcriptional reprogramming within the cerebrovasculature, characterized by enhanced mitochondrial function and reduced inflammatory and senescence-associated signaling.

## Discussion

The present study provides integrated functional, structural, and mechanistic evidence that time-restricted feeding (TRF) preserves cerebromicrovascular integrity and cognitive function during aging. By linking systemic metabolic adaptations to endothelial mitochondrial function within the NVU, these findings identify a mechanistic framework through which dietary timing influences brain aging. Rather than acting solely through systemic metabolic improvements, our data suggests that TRF directly targets microvascular aging processes, positioning the cerebral microvascular endothelium as a key effector of its neuroprotective effects.

Age-related cognitive decline is increasingly understood as a consequence of impaired NVU function [2, 34, 35]. In this context, our findings demonstrate that TRF preserves multiple dimensions of cerebromicrovascular health, including capillary density, NVC, and BBB integrity [36–38]. These structural and functional improvements were accompanied by reductions in neuroinflammation and cellular senescence across multiple cell types within the neurovascular unit. Together, these observations support the concept that preservation of microvascular integrity is a critical determinant of cognitive resilience during aging.

At the mechanistic level, our study identifies endothelial mitochondrial function as a central node linking systemic metabolic state to cerebromicrovascular health. Aging was associated with impaired mitochondrial respiration and reduced metabolic flexibility in cortical tissue, whereas TRF restored mitochondrial bioenergetic capacity. These findings are consistent with the emerging view that mitochondrial dysfunction in endothelial cells contributes to impaired nitric oxide signaling [35, 39], oxidative stress [40], and vascular inflammation [41, 42]. By improving mitochondrial function, TRF appears to enhance endothelial resilience, thereby preserving neurovascular coupling and barrier integrity. An important question arising from these findings is whether the observed reductions in neuroinflammation and cellular senescence are primarily a consequence of preserved BBB integrity or whether they reflect parallel, BBB-independent mechanisms. BBB disruption is a well-established driver of neuroinflammation [43], permitting the extravasation of circulating proteins, immune mediators, and metabolic byproducts that activate microglia and propagate inflammatory cascades [44]. In this framework, the reduced microvascular permeability observed in TRF-treated mice would be expected to limit inflammatory signaling within the brain parenchyma, thereby contributing to the preservation of microglial morphology and function as shown in Figure 4H. However, additional evidence from the present study shows that TRF reduced markers of senescence and altered transcriptional profiles across multiple NVU cell types, including endothelial and non-endothelial populations, suggesting a more global remodeling of the neurovascular environment. In addition, improvements in endothelial mitochondrial function and redox balance provide a plausible cell-intrinsic mechanism through which inflammatory signaling could be attenuated independently of barrier permeability. The observation that ketone supplementation enhances mitochondrial function and reduces endothelial senescence, yet fails to fully recapitulate BBB protection or suppress neuroinflammation, further supports the existence of partially independent pathways. Importantly, the reduction of endothelial senescence by TRF is consistent with emerging evidence that senescent capillary endothelial cells accumulate progressively in the aging mouse brain and represent primary drivers of BBB disruption [45, 46], microvascular rarefaction, and NVC impairment. Recent work using senolytic approaches to deplete these cells has demonstrated restoration of barrier integrity and cognitive function, underscoring that targeting endothelial senescence is a therapeutically meaningful strategy [10]. Our data suggest that TRF achieves a related outcome through metabolic rather than pharmacological means, and may act upstream of senescence induction by restoring mitochondrial redox balance and reducing the genotoxic stress that drives endothelial cell cycle arrest. The pattern of microglial morphological preservation, including maintenance of ramification index and reduced somal hypertrophy, further indicates that TRF attenuates the neuroinflammatory milieu, likely through a combination of BBB-dependent and BBB-independent mechanisms. Transcriptomic evidence for reduced senescence-associated secretory phenotype (SASP) expression across multiple neurovascular cell types supports the interpretation that TRF produces a global anti-inflammatory remodeling of the NVU environment, rather than acting selectively on a single cell type.

A key finding of this study is the identification of circulating ketone bodies as mediators of TRF-induced microvascular benefits. Ketone levels were elevated in TRF-treated animals, and exogenous ketone administration improved mitochondrial function and redox balance in human cerebromicrovascular endothelial cells. Importantly, a ketone-rich diet partially recapitulated the structural and mitochondrial effects of TRF *in vivo*, including improvements in microvascular density and mitochondrial coupling efficiency. However, ketone supplementation did not fully reproduce the effects of TRF on NVC or BBB integrity, suggesting that additional TRF-dependent mechanisms, such as circadian regulation [27] or broader metabolic reprogramming—also contribute to neurovascular protection.

These findings extend prior work linking metabolic interventions to vascular and brain health. Previous studies have demonstrated that TRF reduces oxidative stress and improves vascular function in models of cerebrovascular disease [47], and that fasting-induced metabolic adaptations influence inflammatory and mitochondrial pathways across multiple tissues [48, 49]. Our study advances this field by providing direct evidence that TRF modulates cerebrovascular aging through endothelial-specific mechanisms and by identifying ketone-driven mitochondrial reprogramming as a key component of this effect.

From a translational perspective, these findings suggest that targeting endothelial metabolic function may represent a viable strategy to mitigate microvascular contributions to cognitive impairment and dementia. TRF represents a feasible, non-pharmacological intervention that could be implemented in aging populations [50]. In parallel, pharmacological approaches that mimic fasting-induced metabolic states, such as ketone supplementation or agents that enhance mitochondrial function, may provide scalable therapeutic alternatives. These strategies may be particularly relevant for individuals at risk for vascular cognitive impairment, where microvascular dysfunction is a major driver of disease progression.

Several limitations should be considered. First, while our findings demonstrate strong associations between TRF, mitochondrial function, and cerebromicrovascular health, causal relationships at the cellular level require further investigation. Second, although our single-cell transcriptomic analyses provide insight into cell-type-specific responses, additional studies are needed to define the precise molecular pathways mediating these effects. Third, while ketone supplementation recapitulated several aspects of TRF-induced protection, it did not fully reproduce the neurovascular benefits, highlighting the complexity of systemic metabolic interventions.

In conclusion, this study demonstrates that time-restricted feeding preserves cerebrovascular function and cognitive performance during aging through mechanisms involving endothelial metabolic reprogramming. By linking systemic metabolic adaptations to microvascular and mitochondrial function, these findings provide a conceptual framework for understanding how dietary interventions influence brain aging and identify potential targets for therapeutic strategies aimed at preventing vascular contributions to cognitive decline.

## Methods

### Animals

This study utilized male and female C57BL/6J mice in four groups: 1) young (10-month) control mice (n=20) fed *ad libitum*, 2) aged (24-month) control mice (n=20) fed *ad libitum*, 3) 18-monthold mice (n=20) placed on a time-restricted feeding regimen for 6 months, where they were fed *ad libitum* from 10 a.m. to 4 p.m. and fasted the remainder of the day, 4) 18-month-old mice (n=5) placed on a ketone diet for 6 months. The ketone-rich diet was custom-formulated and obtained from Research Diets and contained 2.7% β-hydroxybutyrate potassium powder. The kcal% of the chow was 22.4% protein, 64.2% carbohydrate, 11% fat, and 2.5% ketone ester. All animals had *ad libitum* access to water and all food, with the exception of the ketone-rich diet, was standard rodent chow. Animals for fasting studies were housed in a reverse light cycle facility (darkness 10 a.m. to 10 p.m.). This study was performed in accordance with approved protocols through the University of Oklahoma Health Science Center (OUHSC) Institutional Animal Care and Use Committee (IACUC) and in compliance with Animal Research: Reporting in Vivo Experiments (ARRIVE) guidelines.

### Radial Arm Water Maze (RAWM)

Mice underwent cognitive testing focused on hippocampal-dependent spatial learning and memory using a radial arm water maze (RAWM). Mice were placed into an 8-arm maze filled with water, where a platform was located in only one arm. Food coloring was added to the maze to obscure the platform, and visual shape cues were located at the end of each arm. Noldus Ethovision software was used for data collection via video tracking. For each trial, mice were randomly placed into one of the arms that did not contain the platform. Mice underwent a learning phase, in which they completed 3 days of trials, with 2 sets of 4 trials per day, allowing up to 60 seconds per trial. 7 days later, their ability to recall the location of the platform was tested during the probe phase, which consisted of 2 sets of 4 trials. The reversal phase took place the following day, when the platform was moved to a new location within the maze. Mice are allowed 4 trials during this period, followed by 4 additional re-learning phase trials. The number of errors, as well as the number of successful escapes by locating the platform were recorded.

### Cranial Window Surgery

Surgical procedures were performed as previously described [51, 52]. To initiate the procedure, mice were anesthetized with 2% isoflurane, and anesthesia was monitored throughout the surgery. Using a stereotaxic frame (51625 W, Stoelting Co, Wood Dale, IL, USA), the head of the mouse was fixed and the body of the mouse was rested on a heating pad (RT-0501, Kent Scientific Corporation, Torrington, CT, USA). In an aseptic environment, the head of the mouse was shaved, and a midline scalp incision was made to expose the skull. Following a 6 mm × 8 mm bi-frontoparietal craniectomy using a high-speed microdrill, the skull plate was removed without disturbing the dura, vessels, and sagittal sinus. To prevent excessive heat or bleeding, ice-cold sterile saline was periodically added to the surgical site. A polymethylpentene window (TPX^™^, 6 mm × 8 mm, thickness 0.125 mm, Mitsui Chemicals) was prepared in advance to fit the size of the craniectomy, which was sterilized and positioned in place of the skull. A biocompatible adhesive (Super Glue by Starbond GAP FILLER, Amazon) was used to secure the window, and sutures (Chromic 5–0 C6 18 5/0 Chromic Gut Suture, Reliable Dental Supply, Fort Worth, TX, USA) were applied to the skin. For optimal recovery, mice were treated with buprenorphine extended release (1 mg/kg, ZooPharm, WY, USA) and Baytril 2.27% (10 mg/kg, Elanco Animal Health, IN, USA) daily for 4 days following the procedure.

### Two-Photon Microscopy

Imaging with two-photon microscopy was performed as described in our previous study [53]. Mice were anesthetized with isoflurane, the head was fixed in a stereotaxic frame, and the body of the mouse was rested on a heating pad. Mice were positioned under a Fluoview FV1000 two-photon microscope with a water immersion 25X objective (Olympus, Tokyo, Japan) using a MaiTai HP DeepSee-OL 690- to 1040nm laser (Spectra-Physics, San Jose, CA). Wheat germ agglutinin (WGA-AF594, ThermoFisher Scientific, MA, USA) was retro-orbitally injected (1mg/mL, 4uL/g body weight) and 5 μm z-intervals were imaged for a baseline visualization of the vasculature. FITC-conjugated dextrans (40kDa (D1845, ThermoFisher Scientific, MA, USA), 10kDa (D1821, ThermoFisher Scientific, MA, USA), (3kDa (D1821, ThermoFisher Scientific, MA, USA), and sodium fluorescein (0.3kDa, Sigma-Aldrich)) were injected retro-orbitally by descending molecular mass. After injection of each tracer, a 15-minute z-stack was imaged. Images were analyzed using ImageJ, as previously reported [10]. Time-stacks from the baseline and after each injection were concatenated to produce a time-z-stack. The “Correct 3D Drift” plugin was utilized to align the images. The first 50 μm (meningeal vessels) were excluded, and the images were converted to maximum-intensity projections. The Trainable Weka Segmentation plugin classified the vasculature in order to create binary vascular masks. The green channel was used to quantify changes in fluorescence outside the vascular area by subtracting the maximal-intensity projection and the vascular masks. Cumulative fluorescence changes (I [a.u.]) relative to baseline (I0 [a.u.]) were plotted over time, where the area under the curve (AUC) represents BBB permeability (I/I0).

### Laser Speckle Contrast Imaging

Laser speckle contrast imaging was performed as previously described [54]. Mice were anesthetized (using 4% isoflurane at induction and 1% maintenance), endotracheally intubated, and ventilated (MouseVent G500, Kent Scientific, Torrington, CT). Animals were placed on a heating pad and body temperature was monitored using a rectal thermometer. Animals were kept with the head fixed in a stereotaxic frame. Using laser speckle contrast imaging (Perimed, Sweden), baseline cerebral blood flow was measured over the somatosensory whisker barrel cortex. Whiskers were stimulated for 30 seconds on alternating sides, with 30 second rest periods in between. Changes in cerebral blood flow from baseline were recorded from the contralateral cortex.

### Functional Ultrasound Imaging and Ultrasound Localization Microscopy (fUS-ULM)

fUS-ULM was performed as previously reported [51, 52]. Mice were anesthetized with isoflurane, the head was fixed in a stereotaxic frame, and the body of the mouse was rested on a heating pad. Iconeus One ultrasound imaging system (Iconeus One – 256 channels, ICONEUS, France), equipped with a 15-MHz MultiArray transducer made of four 1D linear 64 element arrays (IcoPrime-4D MultiArray, ICONEUS, France), was utilized. A sterile suspension of microbubbles (DEFINITY, Lantheus, Billerica, MA, USA) was activated via 45 second agitation. Following retro-orbital injection with 50 μl microbubbles, raw ultrasound images were collected for 10 min at 1000 Hz. Sequential images corresponded to the coherent sum of 8 different transmission angles (−12°, −8.57°, −5.14°, −1.71°, 1.71°, 5.14°, 8.57°, 12°) at an 8,000 Hz Pulse Repetition Frequency. Iconeus One fUS imager (Iconeus, France) employed with the “Compute SuperLoc” software was utilized to create maximum projections of microbubble trajectories within a coronal brain section during the entire recording. “Display SuperLoc” software was used to generate a microbubble intensity map, a microbubble speed map, and a microbubble direction map.

FIJI v. 1.52p software (Wayne Rasband, National Institutes of Health, USA) open-source software package combined with MATLAB (R2023a version) was utilized for vascular density analysis. Grayscale images were converted to binary images using a MATLAB script, which also displayed the vascular coverage in specific brain regions by examining positive pixels in regions of interest including the cortex, hippocampus, and white matter. Using a MATLAB script, the resting volumetric flow (Q) was quantified by modifying the Poiseuille equation on a pixel-by-pixel scale.

Using the binary vascular masks from ULM images, “Local Thickness” algorithm was performed to determine vessel diameter. fUS measurements produced greyscale speeds maps from displacement of the microbubbles, which was used to estimate blood flow velocity. Together, these were utilized for quantitative CBF maps, specific to cortex, hippocampus and white matter.

### Immunohistochemistry

Mice were anesthetized with 4% isoflurane, followed by transcardial perfusion with phosphatebuffered saline (PBS). Brains were dissected and fixed in 4% paraformaldehyde for 48 hours at 4°C, followed by a sucrose gradient (10%, 20%, 30%), and embedded in OCT. Brains were stored at −20°C until sectioning. Brains were sectioned at 50 μm thickness and mounted on positively charged slides. Slides with tissue sections were allowed to equilibrate at room temperature for 30 minutes prior to washing. Slides were washed twice for one minute with milli-Q H_2_O to remove OCT, followed by a 10-minute incubation in 3% H_2_O_2_, and subsequent washing with twice milli-Q H_2_O for 5 minutes. Slides were incubated in 1% NaBH_4_ for two minutes and washed three times for 5 minutes with milli-Q H_2_O, and twice for 5 minutes with Hank’s PBS. Sections were blocked for 2 hours (10x PBS, 10x fish gelatin, donkey serum, BSA, 20% Triton X-100) at room temperature. Sections were then incubated in primary antibody for 48 hours at 4 °C in a humidified chamber. Slides were washed 6 times for 10 minutes in 1X PBS. The secondary antibody was incubated for 2 hours at room temperature in darkness, then washed 6 times in 1X PBS for 10 minutes. Sections were stained with 1:500 DAPI for 2 minutes, followed by washing twice with 1X PBS. Slides were mounted with ProLong Gold and the coverslip was applied. Leica Stellaris microscope was used for confocal imaging for the slides.

**Table:** 

Vascular	CAT	Concentration
CD31	BD Pharmingen 550274	1:50
Endomucin	EMD Millipore MAB2624	
**Neuroinflammation**		
IBA1	Synaptic System 234009	1:250
CD68	SYS HistoSure HS-460004	1:50
**Senescence**		
p16	Abcam AB211542	1:250
p21	Santa Cruz SC817X	1:250

### Vascular analysis

Images were analyzed using FIJI ImageJ. Tiled images from the tissue section were stitched. A maximum projection image was created from the z-stacks. ROIs were selected from cortex, CA1, CA2, CA3, and dentate gyrus, where vascular staining was identified using trainable Weka segmentation. Images were binarized and skeletonized to calculate the vascular density, vessel branching, and vessel junctions.

### Senescence analysis

Images were analyzed using FIJI ImageJ. A maximum projection image was created from the z-stacks. A machine-learning based threshold was applied to the images, and the images were binarized. The “analyze particles” feature was utilized to find particles ≥3μm. Senescent endothelial cells were identified as DAPI+/CD31/Endomucin+/p16+/p21+.

### Neuroinflammation analysis

Images were analyzed using FIJI ImageJ. A maximum projection image was created from the z-stacks. A threshold was applied to the images, and the images were binarized. An ROI was selected over the soma of individual microglial cells, and the area was measured. The neuroanatomy plugin was utilized for sholl analysis. For each microglial cell, concentric circles at 5μm radial steps were added as an overlay, and each intersection of a microglial projection with the overlayed circles was counted. Additionally, we measured the distance from the soma where the maximum number of intersecting projections occurred and the number of primary branches originating from the soma. The ramification index was calculated as (maximum number of intersecting projections)/(number of primary branches originating from the soma).

### Body Composition

Quantitative magnetic resonance (qMR; Echo MRI Whole Body Composition Analyzer 4-in-1500; Echo Medical Systems, Houston, TX, USA) was used to measure body composition of mice longitudinally. Mice were weighed before the scans, and the average fat and lean mass values of three scans over 5 minutes were utilized for analysis.

### Indirect Calorimetry

Indirect calorimetry was performed by placing mice in individual chambers (IDC, Sable Systems International, Las Vegas, Nevada) where TRF regimen, reverse light cycle, and chow diet were maintained. Outputs include food intake, energy balance, respiratory exchange ratio, and locomotion recorded hourly. Data was analyzed using calrapp.org [55].

### Serum Ketone Quantification

Young and aged control mice were subjected to an acute overnight fast. Blood was collected from mice of all groups at the end of the fasting period, and during the feeding period. Serum was isolated by centrifugation and stored at −80°C. Serum was thawed, and β-hydroxybutyrate and acetoacetate concentration were quantified using colorimetric assays (Sigma, MAK041; Abcam ab180875).

### In Vitro Ketone Treatments

Primary human brain microvascular endothelial cells (HCMVECs; Cat# ACBRI-376) were purchased from Cell Systems (Kirkland, WA, USA). Cells were derived from normal human brain cortex and maintained according to the manufacturer’s instructions. HCMVECs were treated for 24 hours with physiological concentrations (1, 2.5, and 4mM) of β-HB (Sigma-Aldrich, H6501) or acetoacetate (Sigma-Aldrich, A8509) (1, 2.5, and 4mM).

### Seahorse Respirometry

After treatment, changes in mitochondrial respiration were measured using the Agilent Seahorse Bioanalyzer and MitoStress kit (Agilent Technologies, 103015–100). Oxygen consumption rate (OCR) was measured after the sequential addition of 1.5μM oligomycin, 1μM FCCP, 1μM antimycin A, and 1μM rotenone. Measurements were normalized to subsequent readings of Hoechst 33342 (NucBlue Live ReadyProbes Reagent, Thermo Scientific, R37605).

### Nitric Oxide measurements (DAF-FM assays)

4-Amino-5-Methylamino-2’,7’-Difluorofluorescein Diacetate (DAF-FM) was utilized as a fluorescent indicator of NO. According to the manufacturer’s manual (Thermo Fisher, CAT#23844) cells were incubated in 5uM for 30 minutes at 37 °C. For quantification, a plate reader was utilized at 485 nm excitation and 520 nm emission. Readings were normalized to subsequent readings of Hoechst 33342 (NucBlue Live ReadyProbes Reagent, Thermo Scientific, R37605).

### Mitochondrial Membrane Potential

JC-1 (Thermo Scientific, T3168) was utilized as a fluorescent indicator of mitochondrial membrane potential. After incubation with JC-1 according to the manufacturer’s manual, cells were imaged using spinning disk microscopy (super-resolution Nikon CSU-W1/SoRa). As the cationic dye accumulates in the mitochondria, the fluorescence changes from green (525nm emission) to red (590nm emission). The ratio of green to red fluorescence was quantified to indicate the mitochondrial membrane potential.

### MitoSox

Cells were incubated in 5uM MitoSOX Red (Thermo Scientific, M36008) for 15 minutes at 37 °C. Fluorescence was measured at 510nm excitation/580nm emission, according to the manufacturer’s manual to measure the production of superoxide. Afterwards, cells were incubated with Hoechst 33342 (NucBlue Live ReadyProbes Reagent, Thermo Scientific, R37605) for 30 minutes at 37 °C. Fluorescence was measured using at 360nm excitation/460nm emission, according to the manufacturer’s manual. Mitochondria were labeled using MitoTracker (Thermo Scientific, M7514). Fluorescence was measured using a plate reader. Cells were imaged using the super-resolution Nikon CSU-W1/SoRa spinning disk confocal microscope.

### Amplex Ultra Red

According to the manufacturer’s manual (Thermo Scientific, A36006), cells were incubated in 50uM Amplex Ultra Red mixture (including 0.1U/ml horseradish peroxidase (HRP)). Fluorescence was measured at 530nm excitation/590nm emission immediately, 20 minutes, and 40 minutes after the addition of the mixture to quantify the production of hydrogen peroxide. Readings were normalized to Hoechst 33342.

### Oroboros Respirometry for TRF Intervention

Mitochondrial respiration was measured using Oroboros NextGen-O2k (Oroboros Instruments, Innsbruck, Austria) with 2mL chambers at 37°C. Following transcardial perfusion with ice cold 1X PBS, the brains were dissected on an ice block. The brain cortex was dissected and placed in BIOPS (2.77mM CaK_2_-EGTA, 7.23mM K_2_EGTA, 20mM imidazole, 20mM taurine, 50mM K-MES, 0.5mM DTT, 6.56mM MgCl_2_, 5.77 mM ATP, and 15mM phosphocreatine, at pH 7.1), and the mass was recorded, followed by permeabilization in saponin (5mg/mL) for 30 minutes and 3 washes for 5 minutes. The machine was calibrated daily for oxygen concentration and hydrogen peroxide production. Buffer Z (105mM K-MES, 30mM KCl, 1mM EGTA, 10mM K_2_HPO_4_, and 5mM MgCl_2_-6H_2_O) was utilized as the respiration medium, which was supplemented with amplex ultra red, superoxide dismutase, and horseradish peroxidase for fluorometric measurements. The SUIT protocol [56] included the addition of 5mM ADP, 20mM β-hydroxybutyrate, 2mM malate, 1mM acetoacetate, 5mM pyruvate and 10mM glutamate, 10mM succinate, 2.5uM oligomycin, 2uM CCCP, and 5uM antimycin A. Oxygen and fluorometric flux were normalized to the cortical tissue mass. Complete ketolysis was indicated after the addition of β-hydroxybutyrate and malate, after the oxidation of β-hydroxybutyrate by β-hydroxybutyrate dehydrogenase (BDH1) and reduction of NAD+ to enter complex I.

### Oroboros Respirometry for Ketone Intervention

Mitochondrial respiration was measured using Oroboros NextGen-O2k (Oroboros Instruments, Innsbruck, Austria) with 2mL chambers at 37°C. Following transcardial perfusion with ice cold 1X PBS, the brains were dissected on an ice block. The brain cortex was dissected and placed in BIOPS (2.77mM CaK_2_-EGTA, 7.23mM K_2_EGTA, 20mM imidazole, 20mM taurine, 50mM K-MES, 0.5mM DTT, 6.56mM MgCl_2_, 5.77 mM ATP, and 15mM phosphocreatine, at pH 7.1), and the mass was recorded. The tissue was mechanically permeabilized via manual homogenization in Buffer Z (105mM K-MES, 30mM KCl, 1mM EGTA, 10mM K_2_HPO_4_, and 5mM MgCl_2_-6H_2_O), which was used as the respiration medium. The machine was calibrated daily for oxygen concentration. SUIT-008 O2 mt D026 was followed for these experiments.

### Single Cell RNA Sequencing and Analysis

Mice underwent transcardial perfusion with ice-cold PBS for 10 minutes. The brains were collected immediately, where ~25mg cortical tissue was dissected and snap frozen and stored at −80°C. Samples from each group were barcoded, pooled, and processed on the Chromium X platform (10x Genomics) to generate single-cell emulsions. cDNA was amplified, purified, and libraries constructed, quality-checked (Bioanalyzer), and sequenced by MedGenome. Reads were processed with Cell Ranger and imported into Seurat. Ambient RNA was removed with *SoupX*, and quality control excluded low-quality cells (nFeature_RNA >100 & <7500, percent.mt <10). Data were normalized (*LogNormalize*), variable features identified, and dimensionality reduction performed with PCA and UMAP. Doublets were detected with *DoubletFinder* and excluded. Cell types were annotated using *scMRMA* with manual marker inspection, then sub-clustered. Senescence signatures were quantified by *AUCell* using curated gene sets, and expression scores were validated with *AddModuleScore*. Differential expressions were assessed with *FindMarkers* and compared to established senescence gene sets. Visualization was performed using *ShinyCell*.

### Electrophysiology

The experiments were performed on acute brain slices. To collect slices, mice were decapitated and their brains were quickly removed and cooled with oxygenated ice-cold sucrose slicing solution containing (in mM) 240 sucrose, 25 NaCl, 2.5 KCl, 1.25 NaH2PO4, 26 NaHCO3, 10 MgCl2, 2 Sodium Pyruvate, 0.4 Ascorbic acid and 10 D-glucose (oxygenated with 95% O2/5% CO2). Appropriate portions of the brain were trimmed and the tissue block containing the hippocampus was glued onto the stage of a vibrating microtome (HM650, Thermo Fisher Scientific) with the support of agar block. Horizontal brain slices (350 μm thick) were collected from the vibrating microtome in oxygenated cold sucrose slicing solution. The slices were transferred to a holding chamber containing oxygenated artificial cerebrospinal fluid (ACSF) containing the following (in mM): 126 NaCl, 2.5 KCl, 1.25 NaH2PO4, 26 NaHCO3, 1.0 CaCl2, 1.0 MgCl2, 2 Sodium Pyruvate, 0.4 Ascorbic acid, and 10 D-glucose (final pH 7.4). The slices were allowed to recover for at least 1 hour in ACSF at room temperature.

The slice was transferred to and positioned on a P5002A multi-electrode array (Alpha MED Scientific Inc., Osaka, Japan) and contact between the slice and electrodes was secured using a piece of Nylon mesh and a slice anchor harp on top of the slice. The chamber was perfused with oxygenated ACSF at a rate of 2 mL/min, at 32°C. Field excitatory postsynaptic potentials (fEPSPs) were generated in the CA1 region of the hippocampus by stimulating electrodes in the CA1 and CA3 regions of the hippocampus along the Schaffer collateral pathway. Input/output curves (I/O curves) were generated by applying increasing stimulus currents to the pathway from 0 to 100 μA and recording the responses as previously described (1, 2). The threshold stimulus for generating fEPSPs was determined as 40%–50% of the stimulus strength needed to generate the maximum fEPSP amplitude during the I/O curve measurement. The slice was stimulated once every 30 seconds until a stable baseline lasting at least 10 minutes was obtained. Long-term potentiation (LTP) was induced using high-frequency stimulation train of 100 pulses at 100 Hz, applied 3 times with 30-second intervals. We resumed baseline stimulation immediately after high-frequency stimulation and recorded fEPSPs for at least 60 more minutes. Finally, we recorded another I/O curve generated as described above. For all recordings, we used the MED-64 system and Mobius software (Alpha MED Scientific Inc). Potentiation was calculated as the percent increase of the mean fEPSP descending slope (10–90% of peak) after high-frequency stimulation and normalized to the mean fEPSP descending slope of baseline recordings during 3 minutes prior to tetanus.

### Statistical Analysis

GraphPad Prism 10 (La Jolla, CA, USA) was used for statistical analysis. Statistical tests including one-way ANOVA with Tukey’s post hoc test, one-way ANOVA with Dunnett’s multiple comparisons, unpaired t-test, two-way ANOVA with Tukey’s post hoc test, simple linear regression, and log-rank (Mantel-Cox) survival curve comparison were performed, as indicated in figure legends. All data were tested for normal distribution and significance was defined as *p<0.05.

## Supplementary Files

This is a list of supplementary files associated with this preprint. Click to download.

- ExtendedFigures.pdf
